# ShortCake: an integrated platform for efficient and reproducible single-cell analysis

**DOI:** 10.1093/bioinformatics/btaf559

**Published:** 2025-10-23

**Authors:** Ryuichiro Nakato, Luis Augusto Eijy Nagai

**Affiliations:** Laboratory of Computational Genomics, Institute for Quantitative Biosciences, The University of Tokyo, Tokyo 113-0032, Japan; Laboratory of Computational Genomics, Institute for Quantitative Biosciences, The University of Tokyo, Tokyo 113-0032, Japan

## Abstract

**Summary:**

Recent advances in single-cell analysis have introduced new computational challenges. Researchers often need to use multiple analysis tools written in different programming languages while managing version conflicts between related packages within a single workflow. For the research community, minimizing the time spent on environment setup and installation issues is essential. We present ShortCake, a containerized platform that integrates a suite of single-cell analysis tools written in R and Python. ShortCake isolates competing Python tools into separate virtual environments that can be easily accessed within a Jupyter notebook. This enables users to effortlessly transition between various environments, including R, even within a single notebook. Additionally, ShortCake offers multiple “flavors,” enabling users to select container images tailored to their specific needs. ShortCake provides a unified environment with fixed versions of various tools, thus streamlining workflows, reducing setup time, and improving reproducibility.

**Availability and implementation:**

The ShortCake image is available on DockerHub (https://hub.docker.com/r/rnakato/shortcake) and Zenodo (DOIs: 10.5281/zenodo.17116765 and 10.5281/zenodo.17118158). The source code is available on GitHub (https://github.com/rnakato/ShortCake).

## 1 Introduction

Single-cell analysis is a powerful method for exploring cellular heterogeneity, lineage, and spatial information by profiling the transcriptome and epigenome of individual cells (Lareau *et al.* 2019, Stuart *et al.* 2019). The landscape of single-cell analysis is rapidly expanding. Currently, even for single-cell RNA sequencing (scRNA-seq) alone, more than 1800 tools are registered in the scRNA-tools database (Zappia *et al.* 2018). As a result, it has become common to combine multiple tools within a single project, or to test and compare different methods for the same analytical step, while working in multiple programming languages. Managing these heterogeneous environments can pose significant challenges, especially for researchers without a bioinformatics background.

Several pipelines have been developed to facilitate such integrated single-cell data analysis using multiple tools. For instance, the Seurat ecosystem in R and the scverse in Python offer valuable frameworks for single-cell analysis (Virshup *et al.* 2023, Hao *et al.* 2024). However, these ecosystems do not provide environments for both R and Python, and do not solve installation problems. Users often struggle with dependency conflicts, version mismatches, and complex setup procedures when trying to combine various tools for their own custom analysis workflows. Although package managers like Conda (https://anaconda.org/) are helpful, they still have difficulty reconciling conflicting package dependencies. These problems can prevent researchers from carrying out their research ideas.

Another challenge is reproducibility, especially across different computational environments. For instance, identical workflows have produced different sets of detected transcripts on macOS versus Linux (Di Tommaso *et al.* 2017). Improving the reproducibility of previous studies has been a key challenge.

To address these challenges, we developed ShortCake, a Docker-based platform that consolidates various single-cell RNA-seq and ATAC-seq (scATAC-seq) analysis tools in both R and Python environments, as well as correlated command-line tools. ShortCake separates conflicting Python packages into separate virtual environments, each of which is accessible via a Jupyter Notebook kernel. This streamlines the process of switching between tools without exiting an interactive session. ShortCake also provides multiple “flavors” of the image, allowing users to download only the necessary components and conserve computational resources. Additionally, since the ShortCake Dockerfile is publicly available, users can also edit it to build custom images that include any necessary additional tools. This architecture dramatically reduces installation costs and facilitates reproducibility among users and host computers. It lowers the barrier to entry for researchers and promotes reproducibility through standardized environments.

## 2 Design and implementation

### 2.1 Intended users

ShortCake is designed to lower the entry barrier for single-cell analysis by providing a pre-configured environment. It is most helpful for researchers with limited programming experience who cannot easily troubleshoot installation errors, or for those who want to run well-established pipelines with multiple tools described in the literature. However, ShortCake may be less suitable for users who require the newest features of rapidly updated tools or want to compare tools with multiple, strictly managed versions.

### 2.2 Overall architecture

Shortcake is distributed as a Docker image built on an Ubuntu 22.04 base layer. Docker packages software and its dependencies into lightweight containers, ensuring that the same environment runs identically on any host computer. To enable GPU computation, the latest version of Shortcake (v3.4.0) uses the CUDA 11.8.0 runtime and cuDNN 8 for Ubuntu 22.04. R is installed directly in the container image (v3.4.0 ships with R 4.5.1). Python environments are managed with Micromamba (https://github.com/mamba-org/micromamba-releases), and each package in the base environment is version-pinned via an env.yaml file.

ShortCake (v3.4.0) includes over 100 tools for single-cell analysis. These tools cover various steps, including quality control, doublet detection, batch integration, trajectory/velocity inference, spatial analysis, multimodal integration, and network reconstruction. ShortCake also includes reference genomes, gene annotations, and related demo datasets. See Supplementary Table 1, available as supplementary data at *Bioinformatics* online for a complete list.

### 2.3 Jupyter notebook execusion

The recommended workflow is to start ShortCake’s Docker container, launch Jupyter Notebook, and connect through a web browser, e.g.:

docker run --rm -p 8888:8888 rnakato/shortcake \

jupyternotebook.sh

Users can also run the container on a remote server and access it from their local laptop.

ShortCake resolves conflicts among Python-based tools by assigning each tool to its own virtual environment. Tools that invoke other tools internally are bundled into the same environment. For instance, UniTVelo (Gao *et al.* 2022), which depends on scvi-tools (Gayoso *et al.* 2022), shares the same virtual environment.

**Figure 1.**
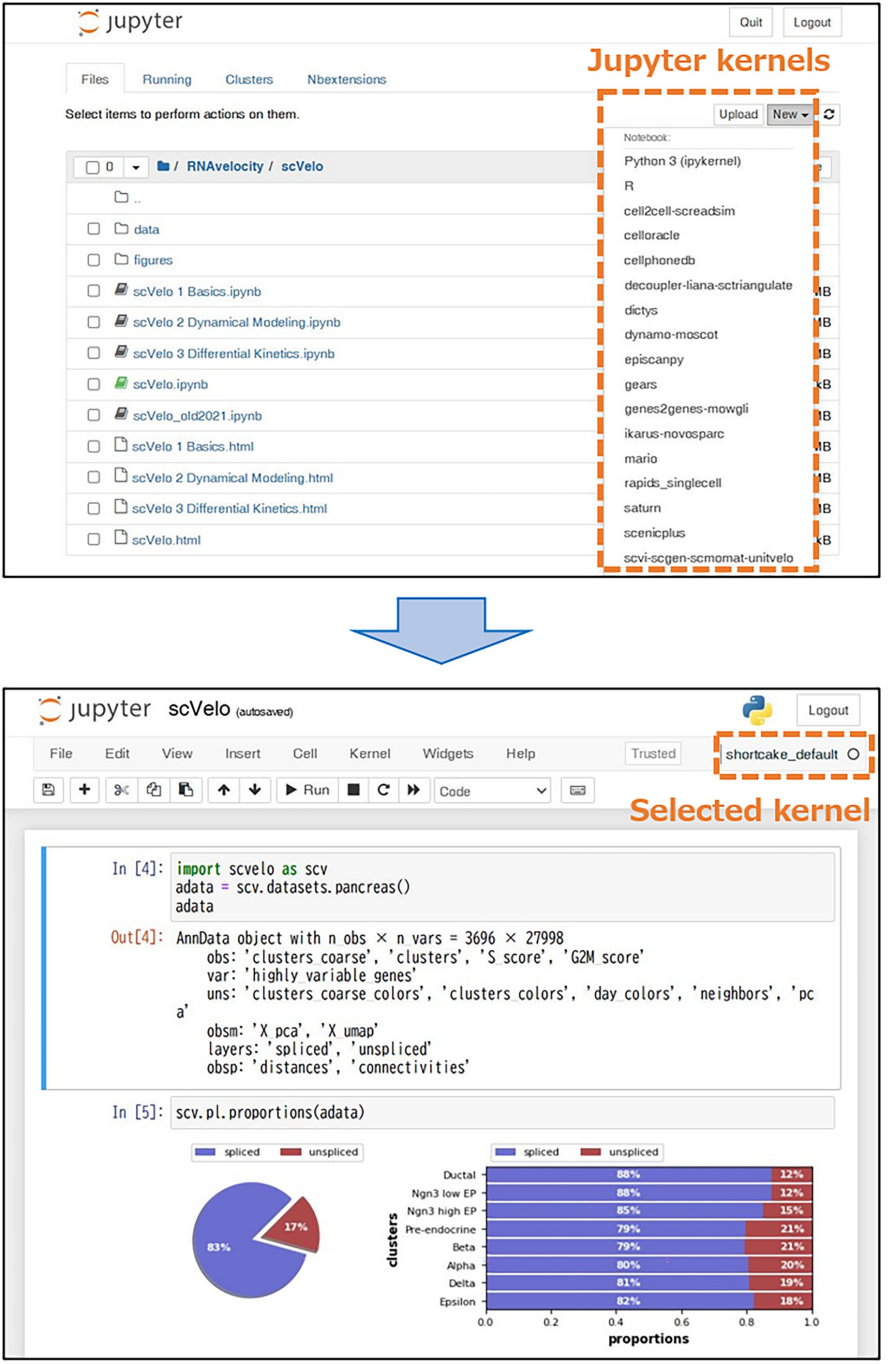
The Jupyter notebook launched from the Shortcake image. Top: Users can select the desired kernel for each virtual environment when creating a new notebook. Bottom: The selected kernel (virtual environment) is activated in the notebook.

To streamline access to the many environments, ShortCake registers a dedicated Jupyter kernel for each, making them selectable within the Jupyter Notebook (Fig. 1). This design enables users to effortlessly switch between environments within a single notebook and facilitates workflows that rely on multiple tools with conflicting dependencies. By clicking the “New” button in Jupyter Notebook and selecting the desired kernel, users can launch an analysis session that runs inside their chosen virtual environment.

### 2.4 RStudio execusion

Although the R environment can also be invoked in Jupyter Notebook, some users may prefer RStudio for R analysis. Shortcake can launch the RStudio Server (https://posit.co/download/rstudio-server/) with this command:

docker run --rm -p 8787:8787 rnakato/shortcake \

rserver.sh 8787

**Figure 2.**
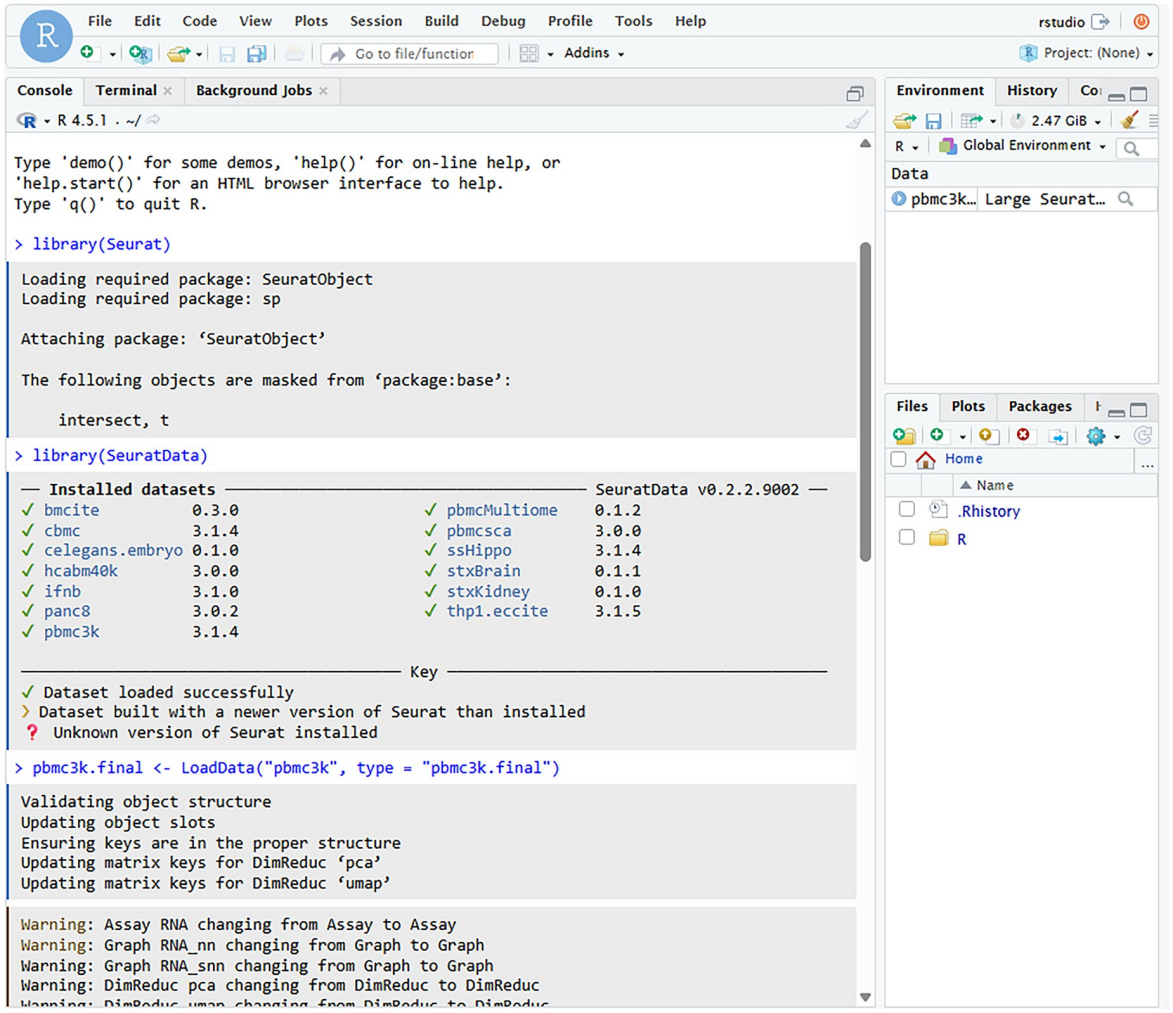
The RStudio Server environment. This example uses Seurat and SeuratData (Hao *et al.* 2024).

Then users can connect through a web browser (Fig. 2). The default username and password are both “rstudio”.

### 2.5 Command-line excusion

Several single-cell tools provide command-line tools. For example, Velocyto (La Manno *et al.* 2018) provides the command velocyto run10x to generate a .loom file. It can be executed as follows:

docker run --rm rnakato/shortcake \

velocyto run10x -m repeat_msk.gtf mypath/genes.gtf

It is also possible to log directly into the ShortCake container and work inside it using the command-line interface.

docker run --rm -p 8888:8888 rnakato/shortcake \

/bin/bash

### 2.6 ShortCake flavors

One drawback of ShortCake is that the Docker image grows rapidly when it contains many virtual environments. Deep-learning tools that require CUDA libraries tend to be particularly large, often adding >10 GB to a single virtual environment. Consequently, the ShortCake image with the full model exceeds 100 GB on Docker, which is impractical for most laptops.

To circumvent this problem, we have prepared several “flavors” of ShortCake. By providing lightweight images that only include the set of tools most users need, it is easier to use for purposes such as tutorials. The main flavors are outlined below:

- shortcake_seurat: Contains only Seurat (Hao *et al.* 2024) and its related packages.
- shortcake_r: Builds on shortcake_seurat with additional R packages. Jupyter Notebook is available, but no Python single-cell tools are installed.
- shortcake_light: Adds the base Python environment to shortcake_r. This flavor bundles Seurat, Scanpy (Wolf *et al.* 2018), Monocle3 (Qiu *et al.* 2017), and scVelo (Bergen *et al.* 2020). This configuration is sufficient for most users.
- shortcake: Extends shortcake_light with nearly all remaining Python virtual environments.
- shortcake_full: The comprehensive image, including every supported tool.

### 2.7 Relation to existing reproducibility platforms

Several well-established platforms have been developed to promote the reproducibility of analysis in the life sciences, including package distribution projects (e.g. BioConda), web-based platforms (e.g. Galaxy), and on-premises high-performance computing portals (e.g. Open OnDemand). Table 1 shows how ShortCake compares to these efforts.

**Table 1.** A comparison with other platforms.

Platform	Pre-built environment	Offline use	Customizability
ShortCake	Yes	Easy	High
Package distribution projects			
(BioContainers/BioConda)	No	Easy	High
Web-based platform			
(Galaxy/Terra/AnVIL)	Yes	Difficult	Low
Computing portal			
(Open OnDemand)	Yes	Difficult	Low

The first key feature of ShortCake is its fully pre-built, local environment. Unlike BioConda, it provides a ready-to-use tool stack that requires no additional installation. In that respect, ShortCake is similar to Galaxy and Open OnDemand, but all computations can run locally, enabling true offline use and avoiding server-side issues such as maintenance and storage limits. Another point is its customizability. While it is usually difficult to modify environments in a web service system, ShortCake images can be modified by editing the original Dockerfile or by creating a new one using ShortCake as the base image. This flexibility enables users to incorporate the latest tools and their own scripts, making it easier to create custom analysis pipelines.

In summary, ShortCake is a lightweight, self-hosted solution that is easier to launch than a Conda and more flexible and offline-friendly than browser-only platforms.

## 3 Discussion

ShortCake has been continuously updated since its inception in 2022 and has been utilized in several studies (Nagai *et al.* 2023, Shibata *et al.* 2024). Its Docker-based approach enables users to replicate an identical analysis environment on any local machine with minimal effort, ensuring reproducible workflows. When Docker privileges are unavailable (e.g. on shared cluster servers), the same image can be run with Singularity (now renamed to Apptainer) (Kurtzer *et al.* 2017) instead.

Community initiatives, such as nf-core (Marques de Almeida *et al.* 2025), have successfully standardized single-cell workflows. These initiatives provide curated Nextflow pipelines that can be executed reproducibly. This type of framework is ideal for large consortia that must run a single, agreed-upon pipeline on numerous samples in a tightly controlled environment. Conversely, ShortCake is designed for individual researchers or small groups exploring suitable case-specific workflows by iterating over alternative tools, parameter settings, and custom scripts. Rather than enforcing a fixed workflow, ShortCake offers a flexible interface that accelerates the prototyping phase for specific biological questions.

Despite its effectiveness, the update issue still remains, especially since maintenance involves more than installing new tools. It requires running each tool’s tutorial from start to finish to confirm that it still works, which often requires additional packages. Automating this process and tracking changes across many tutorials is challenging. The most straightforward solution is to leverage community-driven maintenance on GitHub. If users can open issues and send pull requests whenever a tool breaks, maintainers can quickly identify and resolve issues. It will also be necessary to remove unmaintained tools to keep maintenance costs reasonable. Even in this case, older ShortCake releases still allow users to access the tools. Another option is to evolve ShortCake into a larger database project with a broader contributor base.

As the single-cell research field continues to advance, new technologies will emerge, such as spatial and perturbation analyses. We aim to keep up with these advances by providing the research community with well-validated tools that will facilitate their work.

## Supplementary Material

btaf559_Supplementary_Data
